# Paralysis Agitans

**Published:** 1893-01

**Authors:** Allen J. Smith

**Affiliations:** Galveston; Professor of Pathology in the Medical Department of the University of Texas, Lecturer on Mental and Nervous Diseases in John Sealy Hospital


For Daniel's Texas Medical Journal.

### PARALYSIS AGITANS.

BY ALLEN J. SMITH, M. D.,

Professor of Pathology in the Medical Department of the University of Texas, Lecturer on Mental and Nervous Diseases in John Sealy Hospital.

GENTLEMEN:—The patient illustrative of the discussion into which we are about to enter, is the subject of an affection known as "paralysis agitans." This disease was first systematically described by an English physician, Parkinson, in 1817; and for this reason is not infrequently designated as "Parkinson's disease." The most pronounced symptom of the affection is the tremor, which may be so well noted in this case, which gives origin to the second portion of its common name, "*agitans*"; the first portion of the name, "*paralysis*," being employed because of the loss of muscular power evident in the later stages to a greater or less degree. The disease is also sometimes spoken of as "paralysis festinans," because of the peculiar ten-

---

NOTE.—A clinical lecture delivered before the third year class of the Medical Department of the University of Texas, in John Sealy Hospital, Galveston, Texas.

dency to festination or hastening in the gait of the affected individual.

The etiology of the malady is obscure. It occurs most frequently about middle life, and is nearly equal in its occurrence in the sexes. There is no distinct hereditary tendency in the development of the affection, although occasionally several members of the same generation of a family are affected. Exposure to cold and damp, violent exercise, profound mental emotions, business anxieties, and occasionally peripheral nervous irritations, stand in an apparently causal relation.

The disease may show itself immediately, by the appearance of the characteristic tremors, or, as is not an infrequent feature in the history of individual cases, there may precede this symptom muscular pains, of a more or less general extent, of a sore nature, and often accompanied by cramps. While these cramps and aches are not, as a rule, localized, there is apt to be some one part more affected than the rest; and in this position the tremors are particularly likely to appear. At first these are but slight, intermittent in their manifestation, often only called forth by excitements or fatigue; but eventually they are present almost constantly. Where the disease is gradual in its onset, the tremors are at first quite fine, almost imperceptible, but in the progression of the case become more coarse, although for a large part of the entire extent of the affection they are to a greater or less degree under some voluntary control. Beginning in one or other member, in one hand and arm generally, the tremors distribute themselves gradually over the body, often arranging themselves unilaterally, after the fashion of a hemiplegia; or occurring prominently in one foot and leg, they may pass to the opposite lower member, thus simulating the distribution of a paraplegia. Charcot also describes a crossed invasion, from the arm of one side to the leg of the opposite side. Unless dependent upon some focal disease of the brain, the tremor is not apt to continue localized, but progressively invades the whole body, exclusive of the head and neck. When developed, it is a rapid, rythmical, trembling motion of the trunk and members, rarely absent during the patient's waking hours, and often present as well during sleep. The larger muscle groups, in their tremor, give rise to coarse, rythmical, flapping-like movements, well seen in the arms and hands; but besides these motions, finer, quivering tremors may be distinguished in the individual muscles of the trembling part—tremors within tremors, as it were. These movements are not exaggerated by voluntary efforts at muscular action, but are, on the contrary, for a time at least, suspended temporarily by exercise of the will.

Accompanying, and sometimes preceding the tremor, there are experienced more or less general sensations of muscular soreness and aching, and muscular cramps, which may be constantly present, or may occur only intermittently. Scarcely noted at first, but progressive with the progression of the other muscular symptoms, there comes a peculiar rigidity of the muscles, manifesting itself in the motion and posture of the affected individual. The patient has difficulty in arising to the standing position, and when this is accomplished, the posture is characteristic. The head is carried well forward, the arms are borne flexed at the elbows and at the wrists, the hands usually being held about the iliac regions; the trunk is flexed upon the thighs, and the knees are likewise slightly bent. At the same time, probably as another evidence of the muscular condition, there is assumed a peculiar passive, heavy facial expression, the countenance lighting up poorly, and then quickly resuming its stoic appearance. In connection with the posture described, but probably not distinctly dependent upon it, as is taught by some authorities, there is developed a peculiar gait, which has given rise to one of the names of the disease, the hastening, or festinating gait. One would be disposed to regard this festination, without due consideration, as the result of the body position of the patient. It would seem that with head and body thrown forward, the patient must thrust forth his feet in order to maintain his equipoise and prevent his falling prone; but this forward movement of the limbs urges the body further onward, and the limbs are moved forward more and more rapidly in order to sustain the body. Thus the man, beginning to walk slowly, presently moves more briskly, and eventually breaks into a run, unable to stop without the greatest effort, unless he catch at some permanent object to stay himself, otherwise he falls headlong. That there is, however, some other factor in the production of this symptom than the posture, is suggested by the fact that if one twitch the patient strongly enough, in a backward direction, to cause a slight retrogression, a backward festination may be induced.

With the development of such symptoms, there gradually grows apparent some loss of muscular power, at first slight, but in the advanced stages of the disease sufficiently pronounced to have suggested the term "paralysis" for the affection. This loss of power may, however, be only an apparent loss, the muscular rigidity referred to and the tremor contributing largely to the annullment of muscular movements, reminding one much of the loss of power in spastic spinal paralysis. The existence of this symptom is not uniform, at least in the earlier stages of the affection, and at this period it is often entirely unnoticed. In speaking of the uncertainty of this symptom, I am reminded, too, of the occasional absence of the tremor, which is generally regarded as so characteristic, in which case the pains, muscular soreness and cramps, the posture, facies and gait include the commonly invoked diagnostic features of the disease.

There are rarely any sensory symptoms, unless one may name here the frequent existence of subjective sensations of heat—although for some unexplained reason the thermometric readings in Parkinson's disease are generally above the normal.

The disease is usually divided into three stages by writers, each of variable and generally of prolonged duration. There is thus described a stage of development, such as has been outlined, which is followed by a stationary period, one in which the symptoms of the disease increase but little, and in which there may even occur intervals of amelioration. Eventually, however, if the patient continue to live, the disease passes on with the greatest of uniformity, into a third stage. Here the slight loss of power has become decided, the patient bed-ridden, and sometimes even without the use of the hands and arms. The tremors are constantly present, at least during the waking hours, in their fullest development; the muscles of the neck, the throat and tongue are not infrequently involved, and as a consequence impairments of the speech, mastication and swallowing may present themselves as symptoms. The special senses are not involved. The mind may be unchanged, but in some instances the mental faculties become progressively impaired. The functions of the bladder and rectum may be overthrown; and in its utter helplessnes the average advanced case presents a truly pitiable condition.

The patient may remain in this deplorable state for an indefinite time, awaiting the gradual dissolution of all his powers, or reaching a termination of his woes by the development of a bulbar palsy, or the occurrence of some intercurrent affection. The impairment of nutrition, consequent upon the confinement and the various other depressive influences, contributes freely to induce this latter contingency; and not infrequently an acute pneumonia or phthisis brings the closing passages of the scene.

No distinct knowledge is possessed as to the pathology of the affection. Various organic lesions are described as having been found, but are without any uniformity; and in the absence of any assured knowledge, I purposely refrain from discussing any of the theoretical views advanced. I may say, however, that the lesions described are in general of a sclerotic or degenerative character, and are located in the cord and in the brain.

After this presentation of the subject, I am in position to refer your attention, for illustration of my remarks, to the patient before us:

He is a white man, A. C., aged 38 years, a native of Galveston, and was admitted to the wards of Sealy Hospital, on December 8, 1872. He is single; a machinist by occupation. There is no point in his family history suggestive in relation to the malady in question. His father died of small-pox; his mother of tuberculosis. The patient was one of eight children, two of whom are dead, from pneumonia and typhoid fever, respectively. A brother is now in the hospital because of a chronic leg ulcer, said to be subject to severe tic douloureux, and to leave a strong idiosyncrasy against iodoform. Before the symptoms of the present disease manifested themselves, the patient had never experienced a severe or serious illness. He acknowledges former excesses in venery, alcohol and tobacco; but denies ever having had any veneral disease. He has been much exposed from the demands of his work.

About three years since, after several days of severe labor in an exposed place, he noticed the frequent occurrence of cramps in the right foot, and shortly afterwards experienced a dull, sore pain in the right hip and in the lumbar region. For almost six months these symptoms had existed in variable intensity and frequence, when he noticed that occasionally there were tremors in these parts. At first the tremors were present only after excitements or great fatigue, disappearing spontaneously in a few minutes, and was easily stopped by effort of the will, when his attention was directed to the trembling part. Shortly after the development of the tremors in the right limb, the same sore muscular sensation, followed some time later by tremors, appeared in the arm of the same side. The left arm, and after it the left limb were similarly invaded; and finally, the muscles of the neck, tongue and lower jaw were involved.

As the patient stands before you, the tremor is his most apparent symptom, the entire body exhibiting a rhythmical motion, communicated from the tremor of the limbs. The arms, the forearms and hands move with a coarse, regular, flapping motion; the thumbs and fingers move in a peculiarly regular manner that recalls to me the movements of rolling a pencil or other small object between the thumb and fingers. As an anomalous symptom, I would call your especial attention to the tremors in the muscles of the neck, the tongue and the lower jaw. As may be seen, the whole head shakes from the vibratory action of the neck muscles; and the mumbling speech you have heard is dependent upon the involvement of the muscles of the tongue and lips. He complains of the frequent necessity, in eating, to move the food about in his mouth with his fingers, the tongue being especially unequal to removing the food lodged in the arch of the palate. The tumor of the masseter muscle may be easily recognized by noting the movements of the lower jaw, or by inspecting the masseteric region of the cheeks, just anterior to the ear, where the tremors are distinctly visible. Many authorities deny the existence of tremors in these places in paralysis agitans, and there is no question as to their being generally absent; but slight tremors of the tongue are not infrequent in advanced cases, nor are tremors of the neck muscles very rare. Tremors are, however, quite uncommon about the muscles of the jaw and face; and in this particular, the case is somewhat anomalous. Absent during sleep, these tremors begin daily, shortly after the patient awakes, and continue during his waking hours, whether he be at rest or in motion. He states, that when he is very quiet, and his thoughts wander from himself and his malady, the tremors are of a finer rhythm, although they do not disappear. Excitement, as evinced in discussing his symptoms before him, and high-pitched sounds, as I am able the show by the variations of the hum of a Faradic instrument, exaggerate the intensity of the tremors. At first, by an effort of the will, he could suspend them, and even at present he is able to write in a clear, and almost fine hand. He has been able to work as a machinist up to the date of his admission to the hospital, in filing and polishing the small machinery about sewing machines. Nevertheless, while he is thus able to produce an abeyance of the symptoms in one part of the body, it is not entirely abolished, and may be present in other portions in its usual intensity.

The patient informs me that he notes no difference from the influence of temperature, seasons, or weather.

The flexed position, which I described as assumed by the body in the erect posture, in this affection, is finely exhibited by the patient before you, the head and body leaning forward, the arms a-kimbo, and hands clasped over the hypogastinum and the knees strongly flexed. The festinating gait has never been well marked in this man, giving immediate evidence that festination is not dependent upon the body posture,—else here it would have been well and typically developed. There has been amelioration of this symptom under treatment, however, but even now there is a tendency to break into a rapid and swinging step, when the patient's attention is called from his gait. Ordering the patient to turn rapidly, I am able, moreover, to induce a mild degree of the backward festination, the man being unable to control his backward movements as he turns. This fact, coupled with the evident effort at equilibration in the ordinary anterior festination, as well as the tremors, is strongly suggestive of a cerebellar origin for the disease, which I should be disposed to discuss, were there more support for such a view from post mortem findings. As it is, I prefer but to refer to it as a possible line of explanation of the symptomatology of our case.

The facial expression tallies closely with the description I have given you, the lower part of the face being particularly inert and expressionless. When the man first came under our care, the muscular paretic condition was much more marked than it now is. He was unable to rise without aid, from a chair or bed, to the erect position, although at present these movements are easy of accomplishment. The laxity of the hand grasp, and the slight muscular resistance of the arm to weight exhibit, however, the absence of his natural strength. He continues to complain of a dull, sore, aching pain in the left sacral region, which pain, he states, has been present, at intervals of long duration, for a number of months. He presents no sensory anomalies other than the sensations of heat I have referred to; but there has been an actual increase in the body temperature curve upon his chest, varying between 99.5° and 102° F. I am unable to indicate to you any organic cause for this temperature disturbance, examination of the thoracic and abdominal organs giving no clue. The special senses are in no apparent way involved.

The sexual power began to fail early in the course of the case, and at present both power and appetite are practically abolished

As a rule, in paralysis agitans, there are no abnormalities of the reflex phenomena, but here, as I am able to demonstrate, the knee-jerk of both sides is increased, particularly on the right side, the side first exhibiting the symptoms of the affection. The general functions of the body, apart from the symptoms narrated, are apparantly normal.

In the construction of a diagnosis of paralysis agitans in this instance I have then the following symptom group ; a tremor rythmical in occurrence, almost universal in distribution, constant during the wakiug hours, and diminished by volition and volitional movements ; a peculiar flexed and fixed attitude of the body in the erect position together with a fixed and impressive countenance, and an altered mode of speech, a peculiar manner of locomotion of the type termed "festinating" ; a certain degree of loss of muscular power, the existence of dull, aching pains in different parts of the body, and finally a number of minor phenomena, such as slight fever, increase of patellar reflex, and loss of sexual power and appetite.

The prominence of the symptomatic tremor leads one to regard it as an important basis for differentiation. Tremor is an oscillatory movement produced by the more or less rythmical action of opposed muscles or groups of muscles, not always interfering with purposeful muscular efforts. It is met, besides in the disease under consideration, in old age, in various chronic toxæmias as chronic alcoholism, plumbism or mercurial poisoning, in insular cererbo-spinal sclerosis, in general paralysis of the insane and in chorea. The tremor of this last condition it is true can scarcely be mistaken for that of Parkinson's disease, being distinctly a rythmical, irregular and coarse. The movements in chorea are twitching, jerking and irregular, often interfering decidedly with voluntary efforts ; and taken into consideration together with the other aspects of the case can scarcely be regarded as simulating paralysis agitans. The tremor of general paresis of the insane is met with in individuals rarely presenting any of the symptoms simulating paralysis agitans ; they are of a finer type and are less widely distributed, being largely confined to the lips and tongue, and hands. When they do occur more widely distributed, the exaggeration produced by a voluntary effort is a marked feature of difference as well as the difference in degree and rhythm. The general trend of the case, with its mental, motor, sensory and trophic symptoms easily complete the differentiation. It is asserted by some that cases described as examples of Parkinson's disease in which there exists a distinct tremor of the head has been improperly diagnosed, and are in reality instances of insular or disseminated sclerosis. That such tremors of the head, jaw, tongue and neck are present in the case before us there is no doubt, but I should decline for that reason alone to regard the case as one of disseminated sclerosis, since besides the presence of the features of a case of paralysis agitans arguing the existence of this affection, the tremors differ decidedly from those of insular sclerosis in that they are suspended, not exaggerated, by voluntary efforts, they are not especially present about the head and neck as may occur in insular sclerosis, and there are present, excepting the increase of the knee reflex, no symptoms pointing to focal nervous disease besides the tremors and their consequences as described. The toxic tremors and those of old age should present some definite history of habits, occupation or age. They are all especially liable to manifest themselves about the head and arms, and are, without an exception that occurs to me now, increased by volitional effort. In the advanced cases of the tremor of old age there is usually a wider distinction than I have indicated and it is possible that in well developed instances of senility and paralysis agitans there may be considerable difficulty of diagnosis if the question of age may not be invoked. Alcoholic tremors besides presenting the characters suggested are to be recognized further by the fact that they are worse in the morning after a night's abstinence, and are relieved as soon as access to alcohol has been gained.

Having in such wise distinguished the nature of the tremor in the case before us, we may support this symptom by the clearly recognizable factors of diagnosis, the characteristic posture and gait, to the clear conviction that we are dealing with a true, although slightly, anomalous case of paralysis agitans ; and may in conclusion turn our attention to the question of treatment. Herein I must confess, as I am often compelled to confess, that little may be promised. Few cases have done aught but pursue the course outlined, and these moreover are held up to doubt because of their variety. Of the remedies which have been suggested none have accomplished as decided good in my own experience as atropine, the good effects of which are quite patent in this instance. I have used the drug several times in this affection with at least temporary benefit, and am disposed to regard it favorably. I would prescribe the alkaloid—doses of one-hundred-and-twentieth of a grain thrice daily by the mouth for the sake of convenience, increasing the dose as the tolerance of the the case permits. Almost immediately there may be expected some amelioration in the paretic symptoms, the drug apparently overcoming to a greater or less degree the mild spastic condition of the muscles which I have described as underlying the paresis. Its effects are unfortunately not as permanent as might be desired, nor does the improvement progress without limitation. In the case before us I shall order when I am persuaded that no more effect may be expected from the atropine, warm baths daily, with use of the Faradic current, perhaps some such alterative as arsenic, silver or mercury. Strychnine has been advised by numerous writers and good results claimed for it, although I cannot corroborate those claims and regard the drug as unsuited to an affection whose chief symptoms seem to point to au uncontrolled spinal influence. On the contrary in its opposite, eserine, I should expect to meet the fulfillment of more of the requirements of the disease, although I am unable to make any distinct statements as to its practical worth. Tonics of iron and bitters are frequently indicated in complicating depressions of the digestive or circulatory functions ; but the real treatment in any case is almost purely speculative, and aside from the exercise of the most earnest care in diet and in the conduct of the hygenic surroundings, is without a positive indication as is sought in scientific medicine.

